# CircUBAP2 promotes SEMA6D expression to enhance the cisplatin resistance in osteosarcoma through sponging miR-506-3p by activating Wnt/β-catenin signaling pathway

**DOI:** 10.1007/s10735-020-09883-8

**Published:** 2020-05-29

**Authors:** Lin Dong, Fangfei Qu

**Affiliations:** 1 Department of Pharmacy, Yantai Affiliated Hospital of Binzhou Medical University, No. 717 Jinbu Avenue, Mouping District, Yantai, 264000 Shandong China; 2 Department of Special Inspection, Yantai Affiliated Hospital of Binzhou Medical University, Yantai, Shandong China

**Keywords:** Osteosarcoma, CircUBAP2, miR-506-3p, SEMA6D, cisplatin resistance, Wnt/β-catenin

## Abstract

The occurrence of chemo-resistance is an essential reason for the high morbidity of osteosarcoma (OS) patients. Circular RNAs (circRNAs) have been involved in the regulation of chemo-resistance in cancers. Semaphorins 6D (SEMA6D) is abnormally expressed in many cancers. However, the roles of circUBAP2 and SEMA6D in the chemo-resistance of OS are still unclear. Quantitative real-time polymerase chain reaction (qRT-PCR) was used to detect the expression levels of circUBAP2, SEMA6D and microRNA-506-3p (miR-506-3p). The cisplatin resistance and proliferation of cells were evaluated by 3-(4, 5-dimethyl-2 thiazolyl)-2, 5-diphenyl-2-*H*-tetrazolium bromide assay. Western blot analysis was performed to measure the protein levels of Wnt/β-catenin signaling pathway biomarkers and SEMA6D. Also, the apoptosis, migration and invasion of cells were assessed by Flow cytometry and Transwell assays, respectively. Besides, Dual-luciferase reporter assay was used to verify the interaction between miR-506-3p and circUBAP2 or SEMA6D. We found that the expression levels of circUBAP2 and SEMA6D were increased in cisplatin-resistant OS tissues and cells. Knockdown of circUBAP2 inhibited the cisplatin resistance, silenced Wnt/β-catenin signaling pathway, hindered cell proliferation, migration and invasion, and promoted apoptosis in cisplatin-resistant OS cells, all of which could be reversed by overexpression of SEMA6D. MiR-506-3p could be sponged by circUBAP2 and could target SEMA6D. The suppression of miR-506-3p overexpression on the progression of OS cisplatin resistance could be reversed by SEMA6D overexpression, while miR-506-3p inhibitor also could invert the inhibitory effect of circUBAP2 silencing on the progression of OS cisplatin resistance. In conclusion, CircUBAP2 and SEMA6D played active roles in the progression of OS cisplatin resistance through miR-506-3p, which might provide some new ideas for studying the countermeasures of OS resistance.

## Introduction

Osteosarcoma (OS) is a malignant bone tumor that often occurs in teenagers and children (Mirabello et al. 2009; Misaghi et al. 2018). At present, the treatment of OS mainly adopts surgical resection, supplemented by chemo-radiotherapy (Marina et al. 2004). The discovery of cisplatin and other anti-tumor drugs also greatly improved the prognosis of patients (Dasari and Tchounwou 2014; Ghosh 2019). Despite the continuous improvement of treatment methods, the occurrence of chemo-resistance in OS patients severely hinders the treatment process of OS (He et al. 2014; Koberle et al. 2010). Therefore, it is urgent to explore the new mechanism affecting OS cisplatin resistance.

Circular RNAs (circRNAs) are a class of non-coding RNAs characterized by covalently closed loop and have been found in a variety of diseases, including cancers (Kristensen et al. 2019; Shabaninejad et al. 2019; Su et al. 2019). The differentially expressed circRNAs participated in the regulation of chemo-resistance in many cancers (Hua et al. 2019; Zhu et al. 2019). For example, circ_0081143 improved cisplatin resistance in gastric cancer (Xue et al. 2019). Also, circ_0000285 was involved in cisplatin resistance of bladder cancer (Chi et al. 2019). Of course, circRNAs have been shown to be associated with chemo-resistance in OS, such as circ_001569 and circ_0001258 (Zhang et al. 2018; Zhu et al. 2019). CircUBAP2 is a new circRNA discovered in recent years, which has been proved to be highly expressed in ovarian cancer and triple-negative breast cancer (Sheng et al. 2019; Wang et al. 2018). However, its role in OS and whether it is involved in cisplatin resistance of OS are still unclear.

Semaphorins 6D (SEMA6D) is a member of the SEMA superfamily. With the deepening of research, it has been found that SEMA6D is widely upregulated in gastric cancer and esophageal cancer, and participates in the regulation of cancer progression (Cai et al. 2018; Lu et al. 2016; Zhao et al. 2006). Moriarity et al. reported that SEMA6D might function as an oncogene in OS (Moriarity et al. 2015). However, there are few studies on SEMA6D in cisplatin resistance of OS.

At present, circRNAs are believed to contain conserved microRNA (miRNA) targets, which can be used as miRNA sponges to regulate the expression of target genes (Hansen et al. 2013). The purpose of this study was to explore the role of circUBAP2 and SEMA6D in the progression of cisplatin resistance in OS and to search for the miRNAs interacted with circUBAP2 to clarify the relationship between circUBAP2 and SEMA6D. The discovery of circUBAP2/miR-506-3p/SEMA6D axis provided a new target for the study of the cisplatin resistance of OS.

## Materials and methods

### Samples collection

30 cisplatin-responsive OS patients and 30 cisplatin-resistant OS patients were recruited from Yantai Affiliated Hospital of Binzhou Medical University. OS tumor tissues were removed and recorded as the Tumor-responsive and Tumor-resistant groups, respectively. All tissues were stored at − 80 ℃ until use. This study was informed and agreed by all patients and obtained the approval of the Ethics Committee of Yantai Affiliated Hospital of Binzhou Medical University.

### Cell culture

OS cells (U2OS and SaOS-2) were obtained from American Type Culture Collection (ATCC, Manassas, VA, USA). U2OS and SaOS-2 cells were treated with cisplatin at an increasing concentration to establish cisplatin-resistant OS cells (U2OS/CDDP and SaOS-2/CDDP). All cells were cultured in Dulbecco’s modified Eagle’s medium (DMEM; Hyclone, Logan, Utah, USA) containing 10% fetal bovine serum (FBS; Hyclone), 100 U/mL penicillin and 100 µg/mL streptomycin (Invitrogen, Carlsbad, ca., USA) in 5% CO_2_ at 37 ℃.

### Quantitative real-time polymerase chain reaction (qRT-PCR)

Total RNA was extracted using Trizol reagent (Invitrogen). Then, RNA was reverse-transcribed into complementary DNA (cDNA) using Transcriptor Universal cDNA Master (Roche, Basel, Switzerland). QRT-PCR was performed using SYBR Green (Takara, Dalian, China) in Bio-Rad iCycler system (Bio-Rad, Hercules, CA, USA). Glyceraldehyde 3-phosphate dehydrogenase (GAPDH) and U6 were used as internal controls. The primers were as follows: circUBAP2, F 5′-AGCCTCAGAAGCCAACTCCTTTG-3′, R 5′-TCAGGTTGAGATTTGAAGTCAAGAT-3′; SEMA6D, F 5′-CCGTGTAGTATGGGCCTCAGA-3′, R 5′-TCACAACCCACAGATTGCTAGTG-3′; GAPDH, F 5′-ACCACAGTCCATGCCATCAC-3′, R 5′-TCCACCACCCTGTTGCTGTA-3′; miR-506-3p, F 5′-ACACTCATAAGGCACCCTTC-3′, R 5′-TCTACTCAGAAGGGGAGTAC-3′; U6, F 5′-GCAGGAGGTCTTCACAGAGT-3′, R 5′-TCTAGAGGAGAAGCTGGGGT-3′. The relative expression was analyzed using 2^−ΔΔCt^ methods.

### Cell cisplatin resistance and proliferation assays

Both assays were performed using 3-(4, 5-dimethyl-2 thiazolyl)-2, 5-diphenyl-2-*H*-tetrazolium bromide (MTT) Assay Kit (Apexbio, Huston, Texas, USA). For cisplatin resistance assay, cells were treated with cisplatin at different concentrations for 48 h, MTT solution was added for further incubation for 4 h. Then, dimethylsulfoxide (DMSO) was added into cells and cultured for 15 min. The absorbance was measured, and half-maximal inhibitory concentration (IC_50_) was calculated to evaluate the cisplatin resistance of cells at 560 nm. For proliferation assay, cells were treated with MTT and DMSO at a specific point (0 day, 1 day, 2 day and 3 day) after transfection. The absorbance was detected at 490 nm to evaluate the proliferation of cells.

### Plasmid construction and cell transfection

Small interfering RNA (siRNA) against circUBAP2#1/2 (si-circUBAP2#1/2) and its negative control (si-NC), SEMA6D overexpression plasmid (SEMA6D) and its negative control (pcDNA), miR-506-3p mimic and inhibitor (miR-506-3p and anti-miR-506-3p) or their negative controls (miR-NC and anti-miR-NC) were synthesized by Ribobio (Guangzhou, China). All plasmid vectors were transfected into U2OS/CDDP and SaOS-2/CDDP cells using Lipofectamine 3000 (Invitrogen).

### Western blot (WB) analysis

Cells were lysed with RIPA buffer (Beyotime, Shanghai, China) and quantified with BCA Kit (Beyotime). The same amount of protein was separated via sodium dodecyl sulfate polyacrylamide gel electrophoresis (SDS-PAGE) and transferred onto polyvinylidene fluoride (PVDF) membranes (Millipore, Billerica, MA, USA). The membranes were blocked with 5% skimmed milk and then incubated with the specific antibodies against transcription factor 4 (TCF4; 1:1,000, Invitrogen), β-catenin (1:1,000, Beyotime), SEMA6D (1:1,000, Invitrogen) or GAPDH (1:2,000, Invitrogen) overnight at 4 ℃. Following incubated with secondary antibody (1:2,000, Invitrogen) for 2 h, the membranes were treated with enhanced chemiluminescence solution (Beyotime) to detect the protein signals.

### Flow cytometry

After transfection for 48 h, U2OS/CDDP and SaOS-2/CDDP cells were harvested and re-suspended with binding buffer. Annexin V-FITC/PI Apoptosis Assay Kit (Solarbio, Beijing, China) was used to stain the cells. Flow cytometer (Beckman Coulter, Pasadena, ca., USA) was used to assess the apoptotic cells.

### Transwell assay

Cell migration and invasion assays were performed with Transwell chambers with an 8-µm pore size (Corning Inc., Corning, NY, USA), which non-coated and pre-coated with Matrigel (BD Biosciences, San Jose, ca., USA) to detect migration and invasion, respectively. U2OS/CDDP and SaOS-2/CDDP cells were seeded into the upper chambers containing serum-free medium, while the lower chambers were added DMEM containing 10% FBS. After 24 h, cells on the lower chambers were fixed and stained, and then counted under a microscope (Shoif, Shanghai, China).

### Dual-luciferase reporter assay

The sequences of circUBAP2 or SEMA6D 3’UTR containing the miR-506-3p binding sites and mutant binding sites were inserted into the pGL3-control vectors (Promega, Madison, WI, USA) to build wild-type and mutant-type circUBAP2 or SEMA6D 3’UTR (circUBAP2-WT/MUT or SEMA6D-WT/MUT) reporter vectors, respectively. The above reporter vectors were co-transfected with miR-506-3p mimic or miR-NC into U2OS/CDDP and SaOS-2/CDDP cells. Dual-Lucy Assay Kit (Solarbio) was used to detect luciferase activities.

### Statistical analysis

All data were shown as the mean ± standard deviation. Student’s *t*-test or one-way analysis of variance was used for statistical analysis in SPSS17.0 software (SPSS Inc., Chicago, IL, USA). *P* < 0.05 was considered to be statistically significant.

## Results

### CircUBAP2 was upregulated in cisplatin-resistant OS tissues and cells

**Fig. 1:**
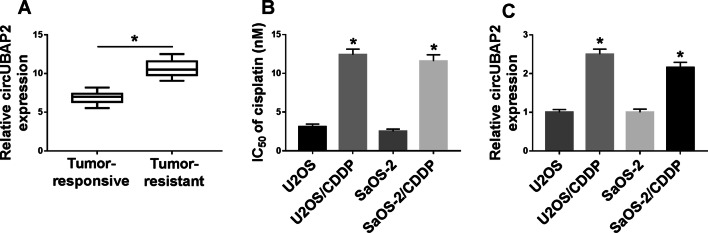
CircUBAP2 was highly expressed in cisplatin-resistant OS tissues and cells. **a** The expression of circUBAP2 was measured by qRT-PCR in cisplatin-resistant OS tissues (Tumor-resistance, N = 30) and cisplatin-responsive OS tissues (Tumor-responsive, N = 30). **b** The cisplatin resistance of OS cells was determined by MTT assay. **c** CircUBAP2 expression was detected by qRT-PCR in OS cells (U2OS and SaOS-2) and cisplatin-resistant OS cells (U2OS/CDDP and SaOS-2/CDDP). *P < 0.05

To evaluate the role of circUBAP2 in OS, we detected the expression level of circUBAP2 in 30 paired of OS tissues (cisplatin-responsive and cisplatin-resistant) using qRT-PCR. Our results revealed that circUBAP2 expression was higher in cisplatin-resistant OS tissues compared with cisplatin-responsive OS tissues (Fig. 1a). Subsequently, we tested the resistance of the constructed cisplatin-resistant OS cells and found that the IC_50_ value of U2OS/CDDP and SaOS-2/CDDP cells was significantly enhanced (Fig. 1b), indicating that the construction of cisplatin-resistant OS cells was successful. In U2OS/CDDP and SaOS-2/CDDP cells, we observed that the expression of circUBAP2 was improved compared with normal OS cells (U2OS and SaOS-2) (Fig. 1c). These data suggested that circUBAP2 might be involved in the cisplatin resistance of OS.

### Knockdown of circUBAP2 restrained the cisplatin resistance of OS cells by silencing Wnt/β-catenin signaling pathway

**Fig. 2:**
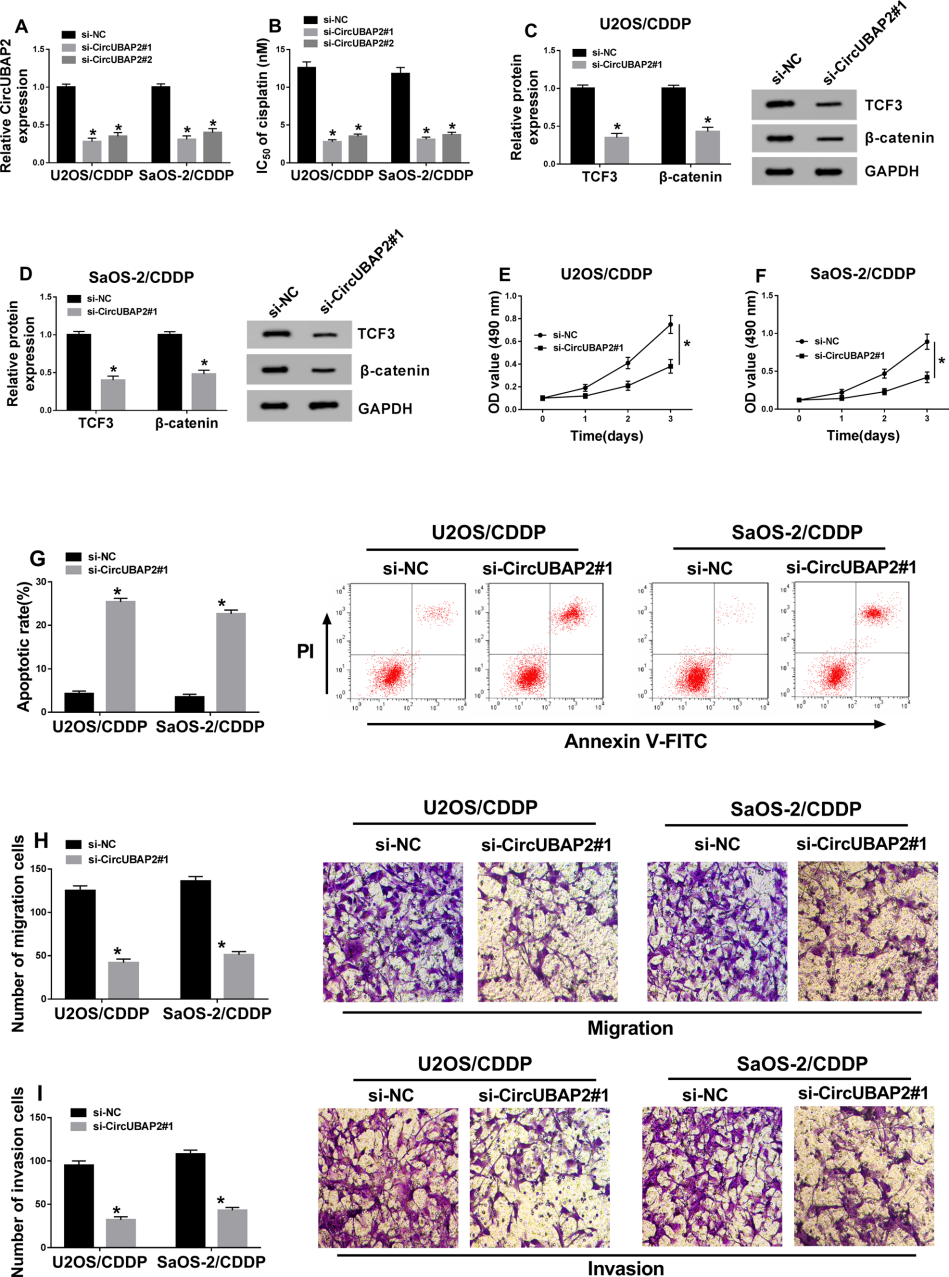
Effects of circUBAP2 expression on the biological function of cisplatin-resistant OS cells. U2OS/CDDP and SaOS-2/CDDP cells were transfected with si-circUBAP2#1/2 and si-NC. **a** The expression of circUBAP2 was detected by qRT-PCR to evaluate the transfection efficiency of si-circUBAP2#1/2. **b** The cisplatin resistance of U2OS/CDDP and SaOS-2/CDDP cells was detected by MTT assay. **c**, **d** The protein levels of TCF3 and β-catenin were detected by WB analysis. MTT (**e**, **f**), Flow cytometry (**g**) and Transwell (**h**, **i**) assays were used to measure the abilities of proliferation, apoptosis, migration and invasion in U2OS/CDDP and SaOS-2/CDDP cells, respectively. *P < 0.05

To explore the biological function of circUBAP2 in cisplatin resistance of OS, we transfected si-circUBAP2#1/2 into U2OS/CDDP and SaOS-2/CDDP cells. Si-circUBAP2#1/2 showed a good transfection efficiency by inhibiting circUBAP2 expression (Fig. 2a), and also significantly reduced the cisplatin resistance in U2OS/CDDP and SaOS-2/CDDP cells, especially si-circUBAP2#1 (Fig. 2b). Through detection the protein levels of TCF3 and β-catenin, we found that silencing of circUBAP2 markedly inhibited the levels of TCF3 and β-catenin in U2OS/CDDP and SaOS-2/CDDP cells (Fig. 2c, d), indicating that circUBAP2 knockdown hindered the activity of Wnt/β-catenin signaling pathway. Besides, knockdown of circUBAP2 could lower the proliferation and improve the apoptosis of U2OS/CDDP and SaOS-2/CDDP cells (Fig. 2e–g). Furthermore, Transwell assay was performed to explore the effects of si-circUBAP2 on the metastasis of OS cells, and the results showed that silenced-circUBAP2 suppressed the migration and invasion of U2OS/CDDP and SaOS-2/CDDP cells (Fig. 2h,i). Hence, all results suggested that circUBAP2 had a positive role in the cisplatin resistance of OS.

### SEMA6D was highly expressed in cisplatin-resistant OS tissues and cells

**Fig. 3:**
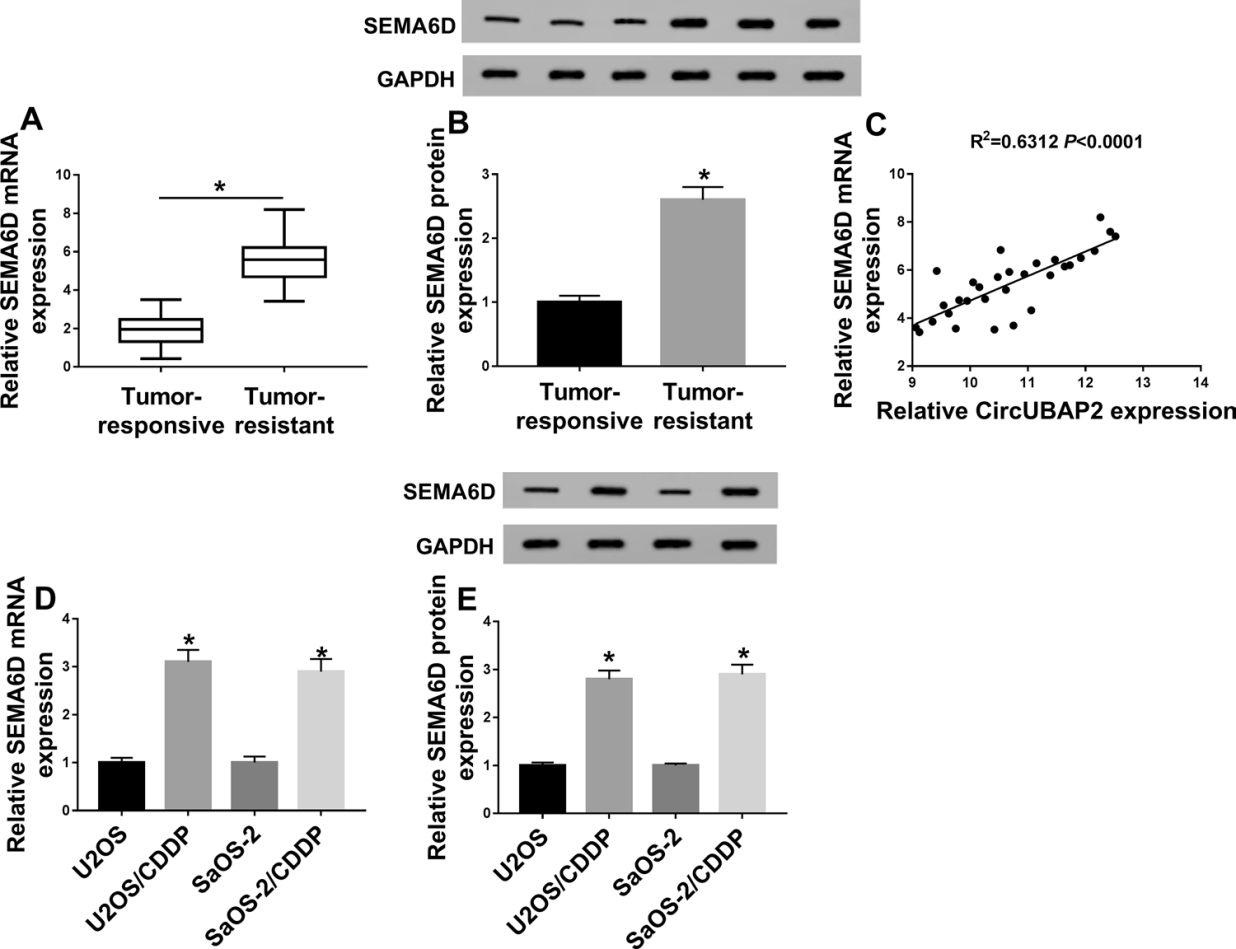
SEMA6D expression was upregulated in cisplatin-resistant OS tissues and cells. **a**, **b** The mRNA and protein expression of SEMA6D were detected by qRT-PCR and WB analysis in cisplatin-resistant OS tissues (Tumor-resistance, N = 30) and cisplatin-responsive OS tissues (Tumor-responsive, N = 30). **c **The correlation between SEMA6D and circUBAP2 was measured by Pearson correlation analysis. **d**, **e** QRT-PCR and WB analysis were used to measure the mRNA and protein expression of SEMA6D in OS cells (U2OS and SaOS-2) and cisplatin-resistant OS cells (U2OS/CDDP and SaOS-2/CDDP). *P < 0.05

On the other hand, we also tested the expression of SEMA6D in OS. As shown in Fig. 3a, we found that SEMA6D expression was increased in cisplatin-resistant OS tissues compared with cisplatin-responsive OS tissues. At the protein level, we get the same results (Fig. 3b). Interestingly, correlation analysis revealed that the expression of SEMA6D was positively correlated with circUBAP2 in OS tissues (Fig. 3c). Besides, compared with U2OS and SaOS-2 cells, we also discovered that the expression of SEMA6D was enhanced in U2OS/CDDP and SaOS-2/CDDP cells (Fig. 3d, e). Therefore, we speculated that the expression of SEMA6D might be related to circUBAP2 in OS.

### Overexpressed SEMA6D reversed the cisplatin resistance of OS cells hindered by circUBAP2 knockdown

**Fig. 4:**
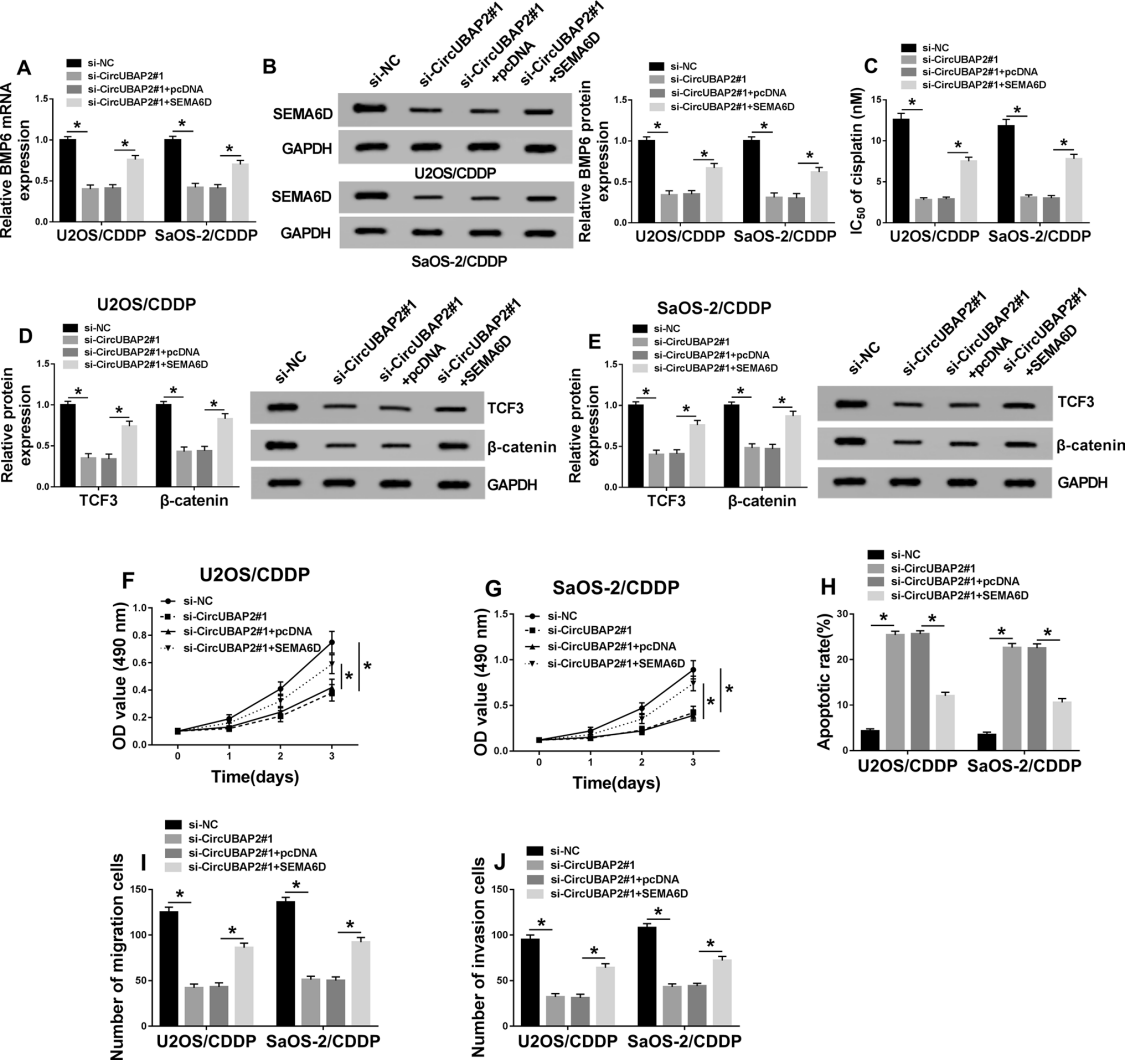
Effects of SEMA6D overexpression on the biological function of cisplatin-resistant OS cells. U2OS/CDDP and SaOS-2/CDDP cells were co-transfected with si-circUBAP2#1 and SEMA6D overexpression plasmid or their negative controls (si-NC and pcDNA). **a**, **b** The mRNA and protein expression of SEMA6D were evaluated by qRT-PCR and WB analysis to assess the transfection efficiency of si-circUBAP2 and SEMA6D overexpression plasmid in U2OS/CDDP and SaOS-2/CDDP cells. **c** The cisplatin resistance of U2OS/CDDP and SaOS-2/CDDP cells was measured by MTT assay. **d**, **e** WB analysis was used to measure the protein levels of TCF3 and β-catenin. MTT (**f, g**), Flow cytometry (**h**) and Transwell (**i,j**) assays were performed to assess the abilities of proliferation, apoptosis, migration and invasion in U2OS/CDDP and SaOS-2/CDDP cells, respectively. *P < 0.05

To determine the role of SEMA6D in OS, we co-transfected si-circUBAP2#1 and SEMA6D overexpression plasmid into U2OS/CDDP and SaOS-2/CDDP cells. Through detecting the mRNA and protein levels of SEMA6D, we found that silenced-circUBAP2 inhibited SEMA6D expression, while the addition of SEMA6D overexpression plasmid increased SEMA6D expression (Fig. 4a, b), indicating that the transfection efficiency of both was excellent and the next experiment could be carried out. Detection of IC_50_ value results revealed that overexpression of SEMA6D could partially reverse the reduction of circUBAP2 knockdown on the cisplatin resistance of U2OS/CDDP and SaOS-2/CDDP cells (Fig. 4c). Besides, through detecting the protein levels of TCF3 and β-catenin, we discovered that SEMA6D improved the activity of Wnt/β-catenin signaling pathway suppressed by circUBAP2 silencing in U2OS/CDDP and SaOS-2/CDDP cells (Fig. 4d, e). Also, MTT, Flow cytometry and Transwell assay results revealed that the suppression effect of silenced-circUBAP2 on the proliferation, migration, invasion and the promotion effect on the apoptosis in U2OS/CDDP and SaOS-2/CDDP cells could be reversed by SEMA6D overexpression (Fig. 4f–j). These results determined the vital role of SEMA6D in the regulation of circUBAP2 on the cisplatin resistance of OS.

### MiR-506-3p was sponged by circUBAP2 and could target SEMA6D in OS

**Fig. 5:**
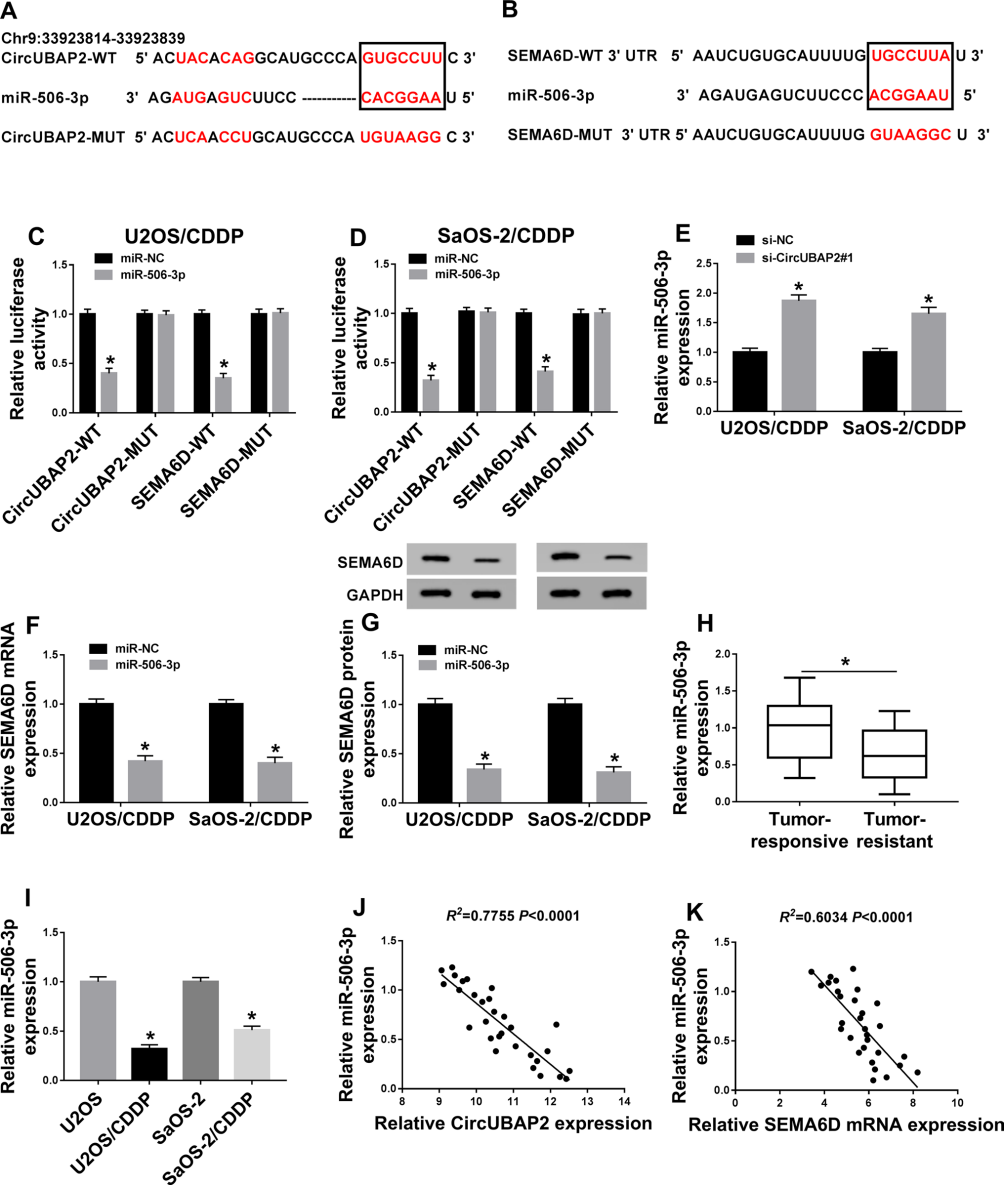
MiR-506-3p was sponged by circUBAP2 and could target SEMA6D in OS. **a**, **b** The predicted target regions of circUBAP2 or SEMA6D 3’UTR containing the miR-506-3p binding sites and mutant binding sites were shown. **c**, **d** Dual-luciferase reporter assay was used to detect the interaction between miR-506-3p and circUBAP2 or SEMA6D in U2OS/CDDP and SaOS-2/CDDP cells. **e** QRT-PCR was performed to measure the effect of circUBAP2 knockdown on the expression of miR-506-3p. **f**, **g** QRT-PCR and WB analysis were used to assess the effect of miR-506-3p mimic on the mRNA and protein expression of SEMA6D in U2OS/CDDP and SaOS-2/CDDP cells. **h**, **i** The expression of miR-506-3p in OS tissues and cells was examined by qRT-PCR. **j**, **k** The correlation between miR-506-3p and circUBAP2 or SEMA6D was measured by Pearson correlation analysis. *P < 0.05

In order to explore the functional relationship between circUBAP2 and SEMA6D, we predicted the miRNAs that could interact with circUBAP2 through the StarBase2.0 tools. It was found that miR-506-3p had complementary binding sites with circUBAP2 (Fig. 5a). Surprisingly, we also found that miR-506-3p could interact with SEMA6D through the Targetscan tools (Fig. 5b). Therefore, we constructed circUBAP2-WT/MUT and SEMA6D-WT/MUT reporter vectors to perform the Dual-luciferase reporter assay. As shown in Fig. 5c, d, miR-506-3p mimic markedly decreased the luciferase activity of circUBAP2-WT and SEMA6D-WT, while had not affect the luciferase activity of circUBAP2-MUT and SEMA6D-MUT in U2OS/CDDP and SaOS-2/CDDP cells. Furthermore, knockdown of circUBAP2 enhanced the expression of miR-506-3p in U2OS/CDDP and SaOS-2/CDDP cells (Fig. 5e). Also, miR-506-3p overexpression could reduce the mRNA and protein expression of SEMA6D in U2OS/CDDP and SaOS-2/CDDP cells (Fig. 5f, g). To verify the function of miR-506-3p, we detected its expression in OS tissues and cells and found that miR-506-3p was lower expressed in cisplatin-resistant OS tissues and cells compared with cisplatin-responsive OS tissues and normal OS cells (Fig. 5h, i). In addition, correlation analysis indicated that the expression of miR-506-3p was negatively correlated with both circUBAP2 and SEMA6D expression (Fig. 5j, k). Therefore, miR-506-3p was a key miRNA between circUBAP2 and SEMA6D.

### MiR-506-3p was involved in the regulation of circUBAP2 and SEMA6D on the cisplatin resistance of OS

**Fig. 6:**
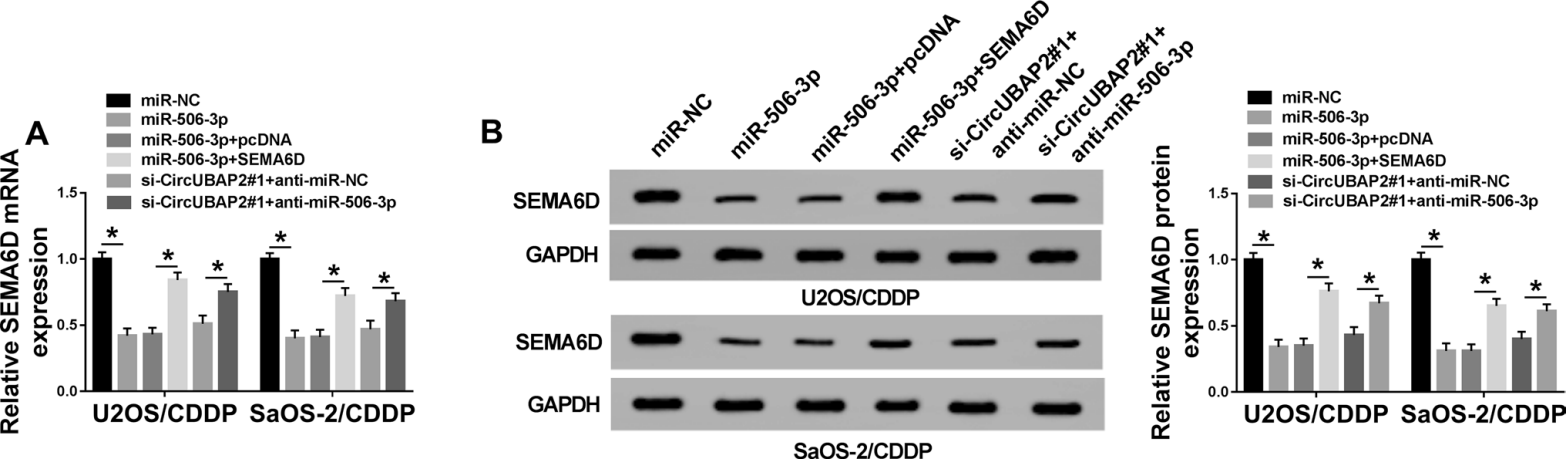
SEMA6D expression was regulated by miR-506-3p and circUBAP2. U2OS/CDDP and SaOS-2/CDDP cells were co-transfected with miR-506-3p mimic and SEMA6D overexpression plasmid or si-circUBAP2#1 and anti-miR-506-3p. **a** The mRNA expression of SEMA6D in U2OS/CDDP and SaOS-2/CDDP cells was measured by qRT-PCR to evaluate the transfection efficiency. **b** WB analysis was performed to detect the protein level of SEMA6D in U2OS/CDDP and SaOS-2/CDDP cells to evaluate the transfection efficiency. *P < 0.05

**Fig. 7:**
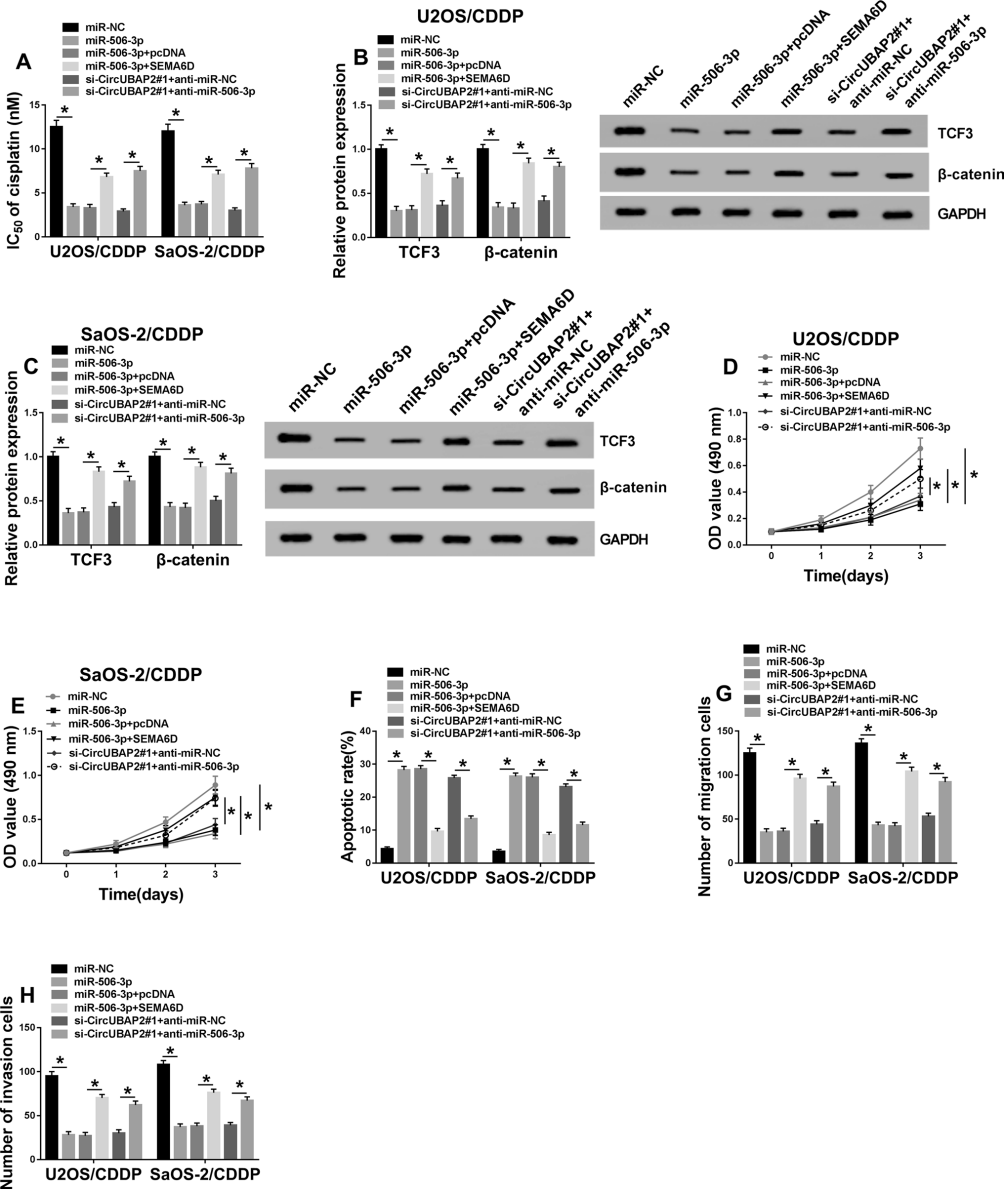
Effects of miR-506-3p expression on the biological function of cisplatin-resistant OS cells. U2OS/CDDP and SaOS-2/CDDP cells were co-transfected with miR-506-3p mimic and SEMA6D overexpression plasmid or si-circUBAP2#1 and anti-miR-506-3p. **a** The cisplatin resistance of U2OS/CDDP and SaOS-2/CDDP cells was evaluated by MTT assay. **b**, **c** The protein levels of TCF3 and β-catenin were measured by WB analysis. MTT (**d**, **e**), Flow cytometry (**f**) and Transwell (**g**, **h**) assays were used to determine the abilities of proliferation, apoptosis, migration and invasion in U2OS/CDDP and SaOS-2/CDDP cells, respectively. *P < 0.05

To further verify the role of miR-506-3p, we co-transfected miR-506-3p mimic and SEMA6D overexpression plasmid or si-circUBAP2 and anti-miR-506-3p into U2OS/CDDP and SaOS-2/CDDP cells, respectively. Firstly, qRT-PCR and WB results showed that the inhibition of miR-506-3p mimic on SEMA6D expression could be reversed by SEMA6D overexpression (Fig. 6a, b). Also, miR-506-3p inhibitor recovered the suppression effect of silenced-circUBAP2 on SEMA6D expression (Fig. 6a, b). These data indicated that the transfection efficiencies of miR-506-3p mimic, SEMA6D overexpression plasmid, si-circUBAP2 and anti-miR-506-3p were excellent. Subsequently, we measured the IC_50_ value of cells and found that miR-506-3p overexpression could hinder the cisplatin resistance of U2OS/CDDP and SaOS-2/CDDP cells, while overexpressed-SEMA6D partially recovered the inhibition of it on the cisplatin resistance of U2OS/CDDP and SaOS-2/CDDP cells (Fig. 7a). Further, the inhibitory effect of circUBAP2 silencing on the cisplatin resistance of cells could also be recovered by inhibiting the expression of miR-506-3p (Fig. 7a). WB results revealed that miR-506-3p overexpression impeded the activity of Wnt/β-catenin signaling pathway in U2OS/CDDP and SaOS-2/CDDP cells, while SEMA6D could invert this effect (Fig. 7b, c). Also, miR-503-3p inhibitor could reverse the activity of Wnt/β-catenin signaling pathway suppressed by circUBAP2 knockdown in U2OS/CDDP and SaOS-2/CDDP cells (Fig. 7b, c). In addition, as shown in Fig. 7d–h, miR-506-3p mimic restrained the proliferation, migration, invasion, and enhanced the apoptosis of U2OS/CDDP and SaOS-2/CDDP cells, while SEMA6D could reverse these effect. On the other hand, miR-506-3p inhibitor also reverted the effects of circUBAP2 silencing on the proliferation, apoptosis and metastasis in U2OS/CDDP and SaOS-2/CDDP cells. All data uncovered that miR-506-3p played an essential role in the regulation of circUBAP2 and SEMA6D on the cisplatin resistance of OS through Wnt/β-catenin signaling pathway.

## Discussion

Nowadays, the discovery of circRNAs provided new insights into cancer development and the causes of chemo-resistance. Zhang et al. reported that circ_001569 improved the proliferation and cisplatin resistance of OS (Zhang et al. 2018). This provides a new basis for the study of circRNAs in the exploration of chemo-resistance of cancers. In our research, we found that circUBAP2 was upregulated in cisplatin-resistant OS tissues and cells. Besides, circUBAP2 knockdown markedly hindered the cisplatin resistance, inhibited the proliferation and metastasis, and improved the apoptosis of cisplatin-resistant OS cells. Wnt/β-catenin signaling pathway is a classical signaling pathway associated with cell proliferation, invasion, and differentiation (Clevers and Nusse 2012). Here, we also found that silenced-circUBAP2 repressed the Wnt/β-catenin signaling pathway activity in cisplatin-resistant OS cells. This evidence indicated that high circUBAP2 expression was crucial for the progression of the cisplatin resistance in OS.

Given the up-regulation of SEMA6D expression in a variety of cancers (Cai et al. 2018; Lu et al. 2016; Moriarity et al. 2015; Zhao et al. 2006), we investigated the function of SEMA6D in the cisplatin resistance of OS. In our study, SEMA6D expression was increased in cisplatin-resistant OS tissues and cells and positively correlated with circUBAP2. Through loss- and gain-of-functional experiments, we found that SEMA6D overexpression could significantly restore the inhibitory effect of circUBAP2 knockdown on cell cisplatin resistance and Wnt/β-catenin signaling pathway. Therefore, we confirmed that circUBAP2 regulated the development of the cisplatin resistance in OS by regulating SEMA6D expression.

To determine the function of circUBAP2 as ceRNA, through bioinformatics prediction and experimental verification, we discovered that miR-506-3p could be adsorbed by circUBAP2, at the same time, miR-506-3p could target SEMA6D. MiR-506-3p has been found as a tumor suppressor in many cancers, including prostate cancer and retinoblastoma (Hu et al. 2019; Wu et al. 2018). Results of Liu et al. determined that miR-506 could improve the sensitivity of ovarian cancer to cisplatin (Liu et al. 2015). Also, miR-506-3p has been reported to be related to the proliferation, metastasis, mesenchymal-to-epithelial transition and autophagy of OS (Jiashi et al. 2018; Wang et al. 2019). In this study, overexpressed miR-506-3p reduced the cisplatin resistance of cisplatin-resistant OS cells, restrained the progression and Wnt/β-catenin signaling pathway activity, which could be reversed by overexpression of SEMA6D. For another, inhibition of miR-506-3p could accelerate the cisplatin resistance, progression and Wnt/β-catenin signaling pathway activity in cisplatin-resistant OS cells hindered by circUBAP2 silencing. These indicated that miR-506-3p played a negative regulatory role in cisplatin resistance of OS. All the results demonstrated that the effects of circUBAP2/miR-506-3p/SEMA6D axis in OS cisplatin resistance.

As a malignant bone tumor with a high incidence, the occurrence of chemo-resistance makes the metastasis and recurrence rate of OS patients constantly increase (Lamoureux et al. 2007; Sakamoto and Iwamoto 2008). Therefore, elucidating the mechanism affecting OS chemo-resistance is expected to provide theoretical basis for solving the occurrence of chemo-resistance of OS. At present, molecular targeted therapy has shown great potential in OS chemo-resistance. Our study found that the absence of circUBAP2 could effectively inhibit the cisplatin resistance of OS. In addition, the mechanism by which circUBAP2 regulated SEMA6D by targeting miR-506-3p also suggested that overexpression of miR-506-3p and knockdown of SEMA6D might be effective ways to inhibit OS cisplatin resistance. This provided new theoretical targets for the molecular targeted therapy of clinical OS chemo-resistance.

In summary, we determined that circUBAP2 and SEMA6D had high expression in cisplatin-resistant OS tissues and cells, and circUBAP2 increased SEMA6D expression to promote the cisplatin resistance of OS through sponging miR-506-3p via Wnt/β-catenin signaling pathway. These results might provide new targets for the study of reducing cisplatin resistance in OS.

## Data Availability

The analyzed data sets generated during the present study are available from the corresponding author on reasonable request

## Abbreviations

OS: Osteosarcoma
circRNAs: Circular RNAs
SEMA6D: Semaphorins 6D
qRT-PCR: Quantitative real-time polymerase chain reaction
WB: Western blot
cDNA: Complementary DNA
GAPDH: Glyceraldehyde 3-phosphate dehydrogenase
IC_50_: Half-maximal inhibitory concentration
DMSO: Dimethylsulfoxide
SDS-PAGE: Sodium dodecyl sulfate polyacrylamide gel electrophoresis
PVDF: Polyvinylidene fluoride
