# Correction to: CircUBAP2 promotes SEMA6D expression to enhance the cisplatin resistance in osteosarcoma through sponging miR-506-3p by activating Wnt/β-catenin signaling pathway

**DOI:** 10.1007/s10735-020-09894-5

**Published:** 2020-07-14

**Authors:** Xuelian Gong, Wei Li, Lin Dong, Fangfei Qu

**Affiliations:** 1 Department of Pharmacy, Yantai City Hospital for Infectious Diseases, No. 717 Jinbu Avenue, Mouping District, Yantai, 264000 Shandong China; 2 Department of Pharmacy, Yantai Affiliated Hospital of Binzhou Medical University, No. 717 Jinbu Avenue, Mouping District, Yantai, 264000 Shandong China; 3 Department of Special Inspection, Yantai Affiliated Hospital of Binzhou Medical University, Yantai, Shandong China

## Correction to: Journal of Molecular Histology 10.1007/s10735-020-09883-8

The authors of the article want to add Xuelian Gong and Wei Li as the first author and co-first author as they have greatly contributed to the revision work. The correct author group is given in this Correction.

All the authors agree the decision Xuelian Gong, Wei Li as the first author and co-first author.

